# Consistency of endothelial function across two consecutive oral contraceptive pill cycles

**DOI:** 10.1113/EP092399

**Published:** 2025-02-27

**Authors:** Lindsay A. Lew, Desiree Tugwell, Tess Leavitt, Melanie Vitez, Emily J. Ferguson, Kyra E. Pyke

**Affiliations:** ^1^ School of Kinesiology and Health Studies Queen's University Kingston Ontario Canada

**Keywords:** endothelial function, individual response, oral contraceptive pill

## Abstract

Oral contraceptive pills (OCPs), composed of an active pill (AP; synthetic hormone) and a placebo pill (PP; synthetic hormone‐free) phase, might impact endothelial function across the OCP cycle depending on the synthetic hormone composition (type and dose). Only one study has investigated very low‐dose second‐generation OCP users, finding impaired endothelial function in the AP versus PP phase. No studies have reported individual changes in endothelial function across OCP phases, and no studies have examined repeatability of endothelial function across multiple OCP cycles. Owing to the consistency of synthetic hormone exposure in OCP users, we hypothesized that group and individual flow‐mediated dilatation (FMD) responses to the OCP phase would be consistent across two OCP cycles. Endothelial function was assessed by FMD via Duplex ultrasound in 17 very low‐dose second‐generation OCP users (19 ± 2 years of age) during the AP phase and PP phase for two consecutive OCP cycles. Individual responses were classified using a threshold of ±2 × typical error. There was a main effect of phase such that FMD was lower in the AP versus PP phase (*P* = 0.022; AP = 4.3% ± 1.3%, PP = 5.4% ± 1.4%). Threshold analysis revealed no consistent responders, and there was no relationship between Δ%FMD in cycle 1 and cycle 2 (*P* = 0.220; *r* = −0.314). Overall, these results suggest that exposure to the synthetic hormones in second‐generation OCPs might be detrimental to vascular function, although this was not demonstrated to be a consistent trait‐like response at the individual level over two cycles.

## Abstract (highlights)

- **What is the central question of this study?**
  Are phase changes in endothelial function consistent across two consecutive oral contraceptive pill (OCP) cycles in second‐generation OCP users?
- **What is the main finding and its importance?**
  Endothelial function was impaired consistently during the active (synthetic hormone) versus placebo (no synthetic hormone) pill phase across two OCP cycles. However, there was substantial intra‐individual variability in responses to phase over consecutive cycles. Exposure to the synthetic hormones in second‐generation OCPs might be detrimental to endothelial function, although this was not demonstrated to be a consistent trait‐like response observed at the individual level over two OCP cycles.

## 1 INTRODUCTION

Oral contraceptive pills (OCPs), composed of an active pill [AP; combined synthetic hormone pills of ethinyl estradiol (EE) and progestin] and a placebo pill (PP; synthetic hormone‐free) phase, can impact endothelial function across the OCP cycle (Meendering et al., 2009; Thompson et al., 2011; Torgrimson et al., 2007). Although relatively few studies have compared various OCP formulations (four main generations exist), the impact of the OCP phase on macrovascular endothelial function appears to be dependent on synthetic hormone composition, including progestin type, in addition to dose of EE (Williams & MacDonald, 2021).

The single study investigating very low‐dose (VLD; 20 µg EE/100 µg levonorgestrel) second‐generation OCPs in isolation reported that users demonstrate impaired flow‐mediated dilatation (FMD) in the AP versus the PP phase; however, low‐dose (LD; 30 µg EE/150 µg levonorgestrel) second‐generation OCP users had no changes in FMD across pill phases (Torgrimson et al., 2007). Levonorgestrel appears to antagonize the impact of oestrogens on endothelial function by decreasing NO bioavailability and thus capacity for endothelium‐dependent dilatation (Zerr‐Fouineau et al., 2009). On the contrary, Shenouda et al. (2018) found no change in FMD across OCP phase in a small subsample of second‐generation OCP users. It is possible that the combined participant pool of low‐dose and very low‐dose second‐generation OCP users in the study by Shenouda et al. (2018) limited the detection of significant phase changes in FMD across various generations; however, the between‐study variability in phasic responses might also be a signal that the endothelial response to synthetic hormones is not universal for all OCP users. There are currently no studies reporting individual changes in FMD across OCP phases, and no studies have examined the repeatability of FMD across multiple OCP cycles. Therefore, it remains unknown whether intra‐individual changes in FMD across pill phases are consistent over consecutive OCP cycles.

There is substantial inter‐individual variability in the impact of endogenous sex hormones across the natural menstrual cycle on endothelial function (D'Urzo et al., 2018; Williams et al., 2001). Interestingly, individual FMD responses from the early follicular (EF; low‐estradiol) to late follicular (LF; high‐estradiol) phase of the menstrual cycle are inconsistent across two consecutive menstrual cycles (Liu et al., 2021). Liu et al. (2021) highlight several methodological concerns that are likely to have contributed to the observed variability in the impact of phase on FMD across the two menstrual cycles. Within an individual participant, there was considerable variability in the timing of the LF phase visit in relationship to ovulation between the two menstrual cycles. Furthermore, individual phase changes in estradiol concentrations were highly inconsistent between the two menstrual cycles. The lack of consistency in estradiol exposure might have contributed to the lack of consistency in FMD responses across the menstrual cycles. OCPs offer a more controlled model to assess the impact of hormones on endothelial function, because the dose and timing of synthetic hormone administration are consistent and predictable. Therefore, we speculated that intra‐individual OCP phase changes in FMD would be consistent across two consecutive OCP cycles.

The purpose of this study was to determine the reproducibility of both group and individual changes in brachial artery FMD across two consecutive OCP cycles in monophasic VLD second‐generation OCP users. Given that the impact of OCP phase on FMD might be influenced by the type and dose of OCP (Meendering et al., 2009; Thompson et al., 2011; Torgrimson et al., 2007), a single composition of OCP was selected for this study. Second‐generation OCPs, containing EE and levonorgestrel, are the most commonly used OCPs in Canada (Statistics Canada, 2015). Owing to the consistency of synthetic hormone exposure in OCP users, we hypothesized that group and individual FMD responses to OCP phase would be consistent across these two OCP cycles. This study furthers our understanding of individual endothelial function responses to cyclic OCP pill phases, which is important because consistent intra‐individual changes in endothelial function in response to synthetic hormones might result in long‐term consequences/benefits to cardiovascular health in OCP users.

The specific objectives of this study were to test the hypotheses that: (1) at the group level, VLD second‐generation OCP users demonstrate an impairment in FMD in the AP versus PP phase across two consecutive cycles; and (2) individual second‐generation OCP users demonstrate consistent FMD responses to OCP phase across two consecutive cycles.

## 2 MATERIALS AND METHODS

### 2.1 Ethical approval

All experimental procedures were approved by the Queen's University Health Sciences and Affiliated Teaching Hospitals Research Ethics Board, which conforms to the standards set by the latest revision of the *Declaration of Helsinki* (with the exception that this study was not registered in a database). The ethics approval reference number is 6004461. Volunteers provided written informed consent forms approved by the same board before participating in the present study.

### 2.2 Participants and screening visit

A priori power calculations indicated that a sample size of 13 participants would be needed, based on Torgrimson et al. (2007) detecting a difference in FMD of 1.9% with an SD of 2.2% across the AP and PP phases. We aimed to recruit 20 young, healthy premenopausal OCP users (18–29 years of age) from Queen's University and the Kingston community to account for dropout and the possibility of a smaller effect size. Each participant came to the laboratory for a screening visit to assess their eligibility and to familiarize them with the experimental protocol. The screening visit included a medical screening questionnaire involving detailed questions about their reproductive history and contraceptive use. In addition, an automated blood pressure (BP) assessment, measurement of height and weight, and a scan of their brachial artery were performed, the last of which was to ensure that a clear image of the artery and a strong velocity signal could be obtained. To be eligible for this study, participants were required to be using monophasic, VLD second‐generation OCPs (20 µg EE, 100 µg levonorgestrel) for a minimum of 3 months. Furthermore, participants had a BP of 90–140 mmHg/50–90 mmHg, a body mass index < 30 kg/m^2^, no history of cardiovascular disease, were recreationally active (≤5 h of structured exercise/week) and were non‐smokers.

### 2.3 Experimental design

**FIGURE 1:**
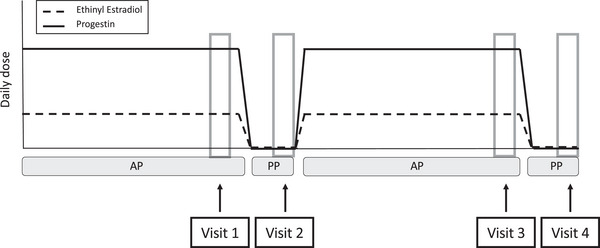
Timing of experimental visits, with one visit in the AP phase and one in the PP phase across two consecutive oral contraceptive pill cycles. The order of visits was counterbalanced between starting in the AP or PP phase. Abbreviations: AP, active pill; PP, placebo pill.

Each participant attended four identical experimental visits over two consecutive OCP cycles. Experimental visits were timed around specific points in the OCP cycle, with each participant undergoing one experimental visit during the PP (no synthetic hormone) phase and one experimental visit during the AP (high synthetic hormone) phase for two consecutive OCP cycles (Figure 1). The placebo visit occurred on days 5–7 of the PP week to ensure the breakdown of synthetic hormones (Johansson et al., 2002). The AP visit occurred on days 5–7 of the third week of APs to ensure the greatest exposure to synthetic hormones. The OCP phase was counterbalanced between participants to start in the active or PP phase of the OCP cycle.

Before each experimental visit, participants refrained from food (>6 h; similar last meal before fast), caffeine (>12 h) and exercise (>24 h), in accordance with current FMD guidelines (Thijssen et al., 2019). All four visits for a single participant occurred at the same time of day (±2 h of each other) to avoid diurnal variation in endothelial function (ter Avest et al., 2005). All four visits occurred 1–3 h following OCP ingestion, because the time to peak concentration of levonorgestrel is 1–4 h following ingestion (Kook et al., 2002). Upon arrival for the experimental visits, participants rested in a supine position for 30 min. Following the rest period, three trials of brachial artery FMD were performed. Each FMD trial was followed by ≥10 min of rest or until arterial diameter and velocity returned to baseline. Following the three FMD trials, a saliva sample was taken to ensure that endogenous oestrogen and progesterone were suppressed by OCP use. Physical activity levels over the week prior were assessed with a Physical Activity Recall Questionnaire (PAR‐Q) (Sallis et al., 1985). Psychosocial stress levels were assessed using the Perceived Stress Scale (PSS) (Cohen, 1988; Cohen et al., 1983).

### 2.4 Experimental procedures

#### 2.4.1 Heart rate and BP

Heart rate (HR; measured in beats per minute) was monitored continuously using a three‐lead ECG. BP was measured six times and averaged (first measure discarded) with an automated sphygmomanometer (BpTRU BPM‐100; BpTRU Medical Devices). BP was taken at the end of each rest period, immediately before the three FMD trials.

#### 2.4.2 Brachial artery diameter and velocity measurements

Diameter measurements of the brachial artery were captured using two‐dimensional ultrasound in B mode (12 MHz; Vivid i2; GE Medical Systems, Mississauga, ON, Canada) with an insonation angle of 68°, as reasoned previously (Pyke et al., 2008). Ultrasound images were recorded with a VGA to USB frame‐grabber (Epiphan Systems Inc., Ottawa, ON, Canada) and saved as avi files using Camtasia Studio (TechSmith, Okemos, MI, USA), as previously described by Jazuli and Pyke (2011). Blood velocity of the brachial artery was measured using Doppler ultrasound operating at 4 MHz (Vivid i2; GE Medical Systems) at an insonation angle of 68° (Pyke et al., 2008). A Multigon 500P TCD (Multigon Industries, Yonkers, NY, USA) was used to determine mean blood velocity, and the signal was recorded for future analysis in LabChart (AD Instruments, Colorado Springs, CO, USA).

#### 2.4.3 Flow‐mediated dilatation

FMD was assessed in the brachial artery. An occlusion cuff was placed on the participant's forearm, distal to the antecubital fossa. Each FMD trial began with 1 min of baseline, followed by 5 min of cuff occlusion at a suprasystolic pressure (250 mmHg) and a subsequent cuff deflation. Arterial diameter and blood velocity were recorded with Duplex ultrasound throughout baseline and the last minute of cuff occlusion through to 3 min following cuff deflation in the brachial artery. All trials of FMD were separated by ≥10 min of rest or until diameter and blood flow returned to baseline. The distance from the antecubital fossa to the ultrasound probe was recorded after the first visit to ensure similar placement of the probe during the following visits. All FMD measures were acquired by a single sonographer.

### 2.5 Data analysis

#### 2.5.1 Hormone analysis

Salivary 17β‐estradiol and progesterone levels were assessed in duplicate by analysis of saliva samples according to the manufacturer's instructions [Salimetrics 17β‐Estradiol (E2) Enzyme Immunoassay Kit and Salimetrics Progesterone (P4) Enzyme Immunoassay Kit, State College, PA, USA]. The total protein concentration of saliva samples was determined using a colorimetric bicinchoninic acid assay (Thermo Fisher Scientific, Waltham, MA, USA).

#### 2.5.2 Mean arterial pressure and HR

Resting mean arterial pressure (MAP) was calculated from automated BPTru (BPTru Medical Devices) systolic and diastolic values using the following equation: MAP = [systolic BP + 2(diastolic BP)]/3. The resting MAP values taken before the three FMD trials were averaged to provide a single baseline MAP value for each experimental visit. Baseline HR was assessed during the 1 min baseline of the FMD trials and averaged to provide a single HR value for each experimental visit.

#### 2.5.3 Arterial diameter and blood velocity

Diameter analysis was conducted by a single observer who was blinded to an experimental visit (PP or AP phase) and cycle (1 or 2). Brachial artery diameter was analysed using automated edge‐tracking software (Encoder FMD and Bloodflow v.3.0.3; Reed Electronics, Perth, WA, Australia), as previously described for the brachial artery (Jazuli & Pyke, 2011). Frame‐by‐frame diameters were compiled into 3 s bins. Blood velocity was analysed using LabChart Software in 3 s time bins, as previously described (Pyke et al., 2008).

#### 2.5.4 Arterial shear rate

The arterial shear rate (SR), a surrogate measure for shear stress without accounting for blood viscosity, was determined in the brachial artery with the following equation: SR = blood velocity/diameter. The SR was quantified during the 1 min baseline and as the area under the curve (SR‐AUC) for 1 min following cuff release, as previously described (Pyke & Tschakovsky, 2007).

#### 2.5.5 Flow‐mediated dilatation

FMD was calculated as an absolute change (AbsFMD) and a percentage change in diameter (%FMD) from baseline to peak diameter post‐deflation (3 s average). In cases where the tracking of the last minute of occlusion was superior and more similar to the tracking post‐deflation (26 of 204 trials), that value was used instead of baseline in the FMD calculation (McGarity‐Shipley et al., 2022; Plotnick et al., 2017; Szijgyarto et al., 2013). The three FMD trials were averaged to produce one FMD value per visit. If FMD trials were discarded owing to a poor‐quality image preventing accurate edge detection (14 of 204 trials), the remaining two trials were averaged to produce a single response value per visit for each participant.

#### 2.5.6 Consistency of vascular responses

Consistency of vascular responses was identified as previously described (Liu et al., 2021). Briefly, threshold‐based dichotomous classification with a threshold of ±2 × typical error (TE) for %FMD was used to classify participants as positive, negative or non‐responders to the OCP phase (Bonafig et al., 2018; Gurd et al., 2016; Hopkins, 2000; Liu et al., 2021; Swinton et al., 2018). The TE was calculated using a separate data set of female participants (*n* = 12), in which FMD was assessed on two separate visits, 3 days apart, during the low‐hormone phase of either the OCP (*n* = 8) or natural menstrual cycle (*n* = 4) (Tremblay et al., 2019). Participants with a change score (AP %FMD − PP %FMD) that fell above the threshold were classified as positive responders, participants with a change score that fell below the threshold were classified as negative responders, and participants who fell between the thresholds were classified as non‐responders (Liu et al., 2021). If participant response classification was the same across both OCP cycles, the participant was considered a consistent responder/non‐responder.

#### 2.5.7 Statistical analysis

Statistical analyses were performed in SPSS v.29 (IBM, Armonk, NY, USA; 2023). Values were expressed as the mean ± SD, and statistical significance was set to *P* < 0.05. Linear mixed models, with factors OCP cycle (cycle 1 and cycle 2) and OCP phase (AP and PP), were used to assess differences in the main outcome variables %FMD and AbsFMD, in addition to other baseline variables, including SR, diameter, HR, BP and salivary E2 and P4. Student's paired *t*‐test was used to assess differences in ΔFMD across phases (AP–PP phase) in cycles 1 and 2. Bivariate correlation analysis was used to determine whether a change in FMD across OCP phases (AP–PP) in cycle 1 was associated with a change in %FMD in cycle 2 and whether a change in endogenous hormones across phases was associated with a change in %FMD across phase. Within‐day reliability of %FMD was assessed using intraclass correlation coefficients (ICC‐ two‐way random effects, absolute agreement, single rater, 95% confidence interval) and classified as follows: <0.50 = poor, 0.50–0.75 = moderate, 0.75–0.90 = good and >0.90 = excellent (Portney, 2020).

## 3 RESULTS

### 3.1 Participant characteristics

Twenty‐two young, healthy individuals using VLD second‐generation OCPs were recruited for this study. Three participants dropped out before initiating the study owing to time commitment. Two participants dropped out before completing all four experimental visits owing to the termination of OCPs. Therefore, a total of 17 participants (*n* = 17) completed this study. Participants were 19 ± 2 years old, with a body mass index of 23.5 ± 3.1 kg/m^2^. Participants were using Alesse (*n* = 7), Alysena (*n* = 9) or Aviane (*n* = 1), all of which had the dosage of 20 µg of EE and 100 µg levonorgestrel for 21 days and a 7 day period without synthetic hormones. The average length of current OCP use was 2.83 ± 1.83 years. All participants self‐identified their sex at birth as female and their gender as female or primarily feminine. Participants self‐identified their race as White (*n* = 15) or East/Southeast Asian (*n* = 2). All participants self‐reported that they had never previously given birth (*n* = 17). There was no effect of phase (*P* = 0.597) or cycle (*P* = 0.969) or an interaction effect (*P* = 0.385) for the 7 day PAR‐Q scores. Likewise, there was no effect of phase (*P* = 0.335) or cycle (*P* = 0.860) or an interaction effect (*P* = 0.999) for the PSS scores.

### 3.2 Estradiol and progesterone

There were no group‐level differences in endogenous estradiol or progesterone between the AP and PP phases in cycles 1 and 2 (Table 1). Results did not change when hormone concentrations were normalized to total protein content (data not shown).

**TABLE 1:** Baseline haemodynamics, flow‐mediated dilatation and hormone measures during the active and placebo phases across two cycles.

Parameter	Cycle 1		Cycle 2		*P*‐value		
	AP phase	PP phase	AP phase	PP phase	Cycle	Phase	Interaction
HR (beats/min)	63 ± 4^a^	59 ± 5	63 ± 6^a^	60 ± 4	0.577	**0.015**	0.531
MAP (mmHg)	77 ± 7	78 ± 7	78 ± 12	78 ± 7	0.981	0.821	0.793
SBP (mmHg)	102 ± 8	102 ± 7	101 ± 8	102 ± 8	0.345	0.436	0.764
DBP (mmHg)	65 ± 7	66 ± 7	64 ± 7	66 ± 7	0.398	0.077	0.995
SR AUC60	3633.0 ± 945.7	3852.6 ± 1064.6	3628.7 ± 991.1	3827.1 ± 1083.4	0.952	0.403	0.966
Baseline diameter (cm)	0.342 ± 0.024	0.336 ± 0.026	0.341 ± 0.028	0.341 ± 0.029	0.799	0.638	0.612
AbsFMD (cm)	0.014 ± 0.007^a^	0.019 ± 0.007	0.014 ± 0.006^a^	0.017 ± 0.007	0.479	**0.016**	0.509
Estradiol (pg/ml)	1.18 ± 0.62	1.16 ± 0.43	0.99 ± 0.37	1.11 ± 0.38	0.286	0.681	0.574
Progesterone (pg/ml)	153.76 ± 116.53	149.79 ± 105.31	144.29 ± 93.52	173.94 ± 147.40	0.798	0.654	0.557

*Note*: Values are presented as the mean ± SD (*n* = 17). Significant findings (*P *< 0.05) are shown in bold. The *P*‐values are presented as a main effect of cycle, phase and cycle × phase interaction.

Abbreviations: AbsFMD, absolute flow‐mediated dilatation; AP, active pill; HR, heart rate; MAP, mean arterial pressure; PP, placebo pill; SR AUC60, shear rate area under the curve for 60 s post‐cuff release.

^a^ Denotes that values are significantly different in the AP phase versus the PP phase (main effect of phase).

### 3.3 Baseline haemodynamics

Baseline haemodynamic variables are presented in Table 1. HR was significantly higher in the AP phase compared with the PP phase in both cycle 1 and 2 (main effect of phase; Table 1). There were no differences in MAP across phases or cycles. All participants had BP values < 120/80 mmHg, with the exception of one participant who was at the low end of the prehypertensive range (122/82 mmHg).

### 3.4 Baseline diameter and FMD

**FIGURE 2:**
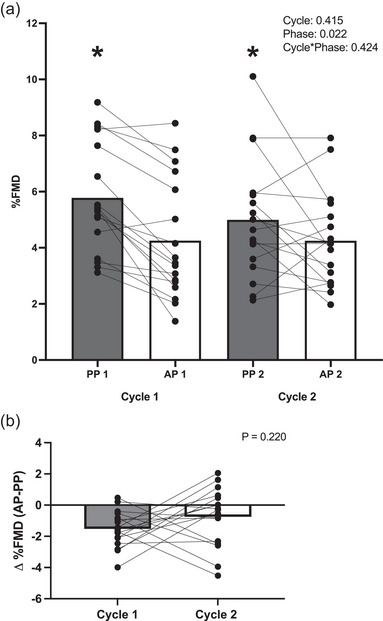
(a) %FMD in the AP and PP phase of two OCP cycles. (b) Cycle Δ%FMD (AP–PP). Bars represent mean responses, filled circles represent individual responses, and lines connect individual AP and PP responses. *Main effect of phase, *P *< 0.05. Abbreviations: AP, active pill; FMD, flow‐mediated dilatation; PP, placebo pill.

Baseline diameter did not change across phases or cycles (Table 1). There was a main effect of phase on %FMD (Figure 2) and AbsFMD (Table 1) such that FMD was significantly higher in the PP phase compared with the AP phase. The change in %FMD (AP %FMD − PP %FMD) was not significantly different between cycles 1 and 2 (*P* = 0.220; Figure 2b). The SR stimulus [shear rate area under the curve for 60 s post‐cuff release (SR AUC60)] did not differ between phases or cycles (Table 1). Results were not altered when FMD was covaried with SR AUC60. Change in endogenous hormones across OCP phases was not related to change in %FMD (ΔE2 and Δ%FMD: *r* = −0.097, *P* = 0.584; ΔP4 and Δ%FMD: 0.228, *P* = 0.194; ΔE2/P4 ratio and Δ%FMD: *r* = −0.166, *P* = 0.350).

**FIGURE 3:**
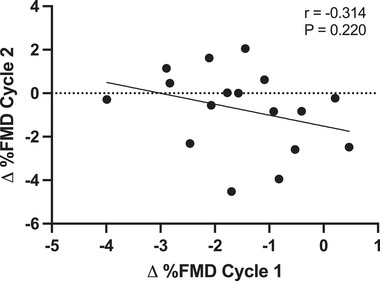
Bivariate correlation between Δ%FMD (AP–PP) in cycle 1 and cycle 2. Abbreviations: AP, active pill; FMD, flow‐mediated dilatation; PP, placebo pill.

**FIGURE 4:**
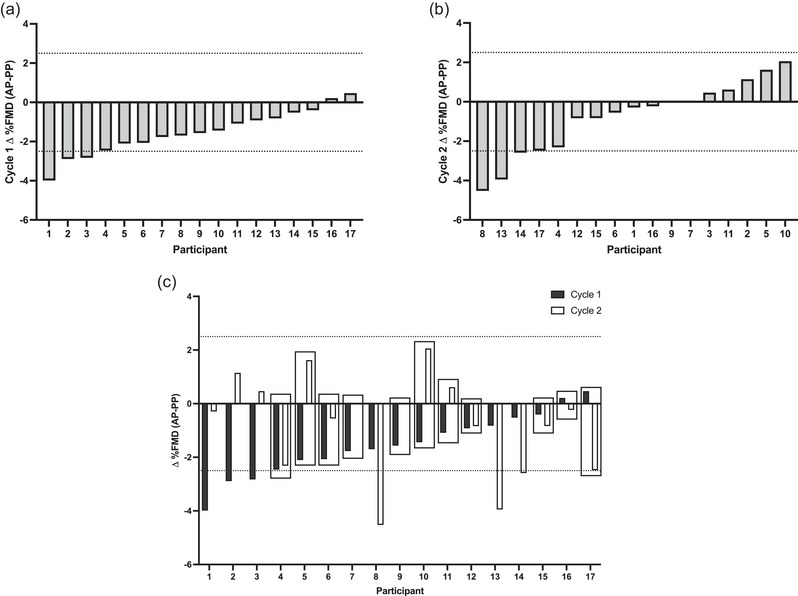
Threshold‐based dichotomous classification of %FMD responses across phases in cycles 1 and 2. (a) Phase changes in %FMD in cycle 1. (b) Phase changes in %FMD in cycle 2. (c) Comparison of individual participant phase changes in cycles 1 and 2. The dotted line represents ±2 × TE. A solid line box indicates consistent non‐responders. Abbreviations: AP, active pill; FMD, flow‐mediated dilatation; PP, placebo pill; TE, typical error.

There was no relationship between Δ%FMD in cycle 1 and cycle 2 (Figure 3). In alignment with the group analysis, the majority of participants had a numerically lower FMD in the AP versus the PP phase (Figure 4a,b); however, there were no consistent responders. Specifically, there were no positive responders (AP > PP) in either cycle. Although there were three negative responders in each of cycles 1 and 2 (AP < PP), none of the negative responders were consistent across cycles. This resulted in six inconsistent responses (35% of the sample inconsistent; Figure 4c). Most response magnitudes failed to cross the threshold in both cycles, resulting in 11 consistent non‐responders (Figure 4c). Within‐day reliability of %FMD (ICC) was an average of 0.78, representing good reliability (cycle 1 AP = 0.79, cycle 1 PP = 0.69, cycle 2 AP = 0.77 and cycle 2 PP = 0.86).

## 4 DISCUSSION

This study was the first to assess conduit artery endothelial function across two consecutive OCP cycles in VLD second‐generation OCP users. The results of the present study found a group‐level decrease in FMD during the AP phase compared with the PP phase over two consecutive OCP cycles. Our results add to the very limited body of research that has explored the impact of a single type and dose of OCP on endothelial function, and importantly, reproduce the single study also demonstrating impairment in endothelial function during the phase of high synthetic hormone exposure in VLD second‐generation OCP users (Torgrimson et al., 2007). However, there was no relationship between change in FMD across phases (AP–PP) in cycles 1 and 2. At the individual level using dichotomous threshold (±2 × TE) classification, although the majority of participants had a lower FMD in the AP phase, there were few individuals with sufficiently large changes with phase to be considered negative responders (*n* = 3 negative responders and *n* = 14 non‐responders for each cycle). Comparing across cycles, six participants (35%) were classified as inconsistent responders (negative responders in one cycle and non‐responders in the other cycle), and the majority of participants (65%) were classified as consistent non‐responders. Aligned with the lack of correlation between phase changes in FMD in cycle 1 and cycle 2, five of the consistent non‐responders had directionally opposite phase changes in each cycle. The small proportion of negative responders to phase and lack of consistency, despite the significant group‐level decrease in FMD, reflects both intra‐individual variability in responses to phase and a small effect relative to our threshold of ±2 × TE. Overall, contrary to our hypothesis and despite a stable dose of synthetic hormones, there was considerable intra‐individual variability in FMD responses to OCP phase.

### 4.1 Impact of OCP phase on FMD

The present study found a group‐level effect of the OCP phase on FMD, such that FMD was lower during the AP versus PP phase across two consecutive OCP cycles. These results are in agreement with those of Torgrimson et al. (2007), who demonstrated a similar decrease in FMD during the AP phase in VLD second‐generation OCP users, and align with cross‐sectional comparisons that revealed lower FMD in second‐generation OCP users compared with naturally menstruating women (Franceschini et al., 2013; Lizarelli et al., 2009). On the contrary, FMD has been shown to increase during the AP phase of third‐ and fourth‐generation OCP users (Meendering et al., 2009; Thompson et al., 2011), and no effect of phase has been reported in LD second‐generation OCP users (Torgrimson et al., 2007). This suggests a negative effect on FMD specific to VLD second‐generation OCP use.

Unopposed EE was shown to increase FMD (Torgrimson et al., 2007), potentially occurring through an increase in endothelial NO synthase expression and thus, NO bioavailability (Kleinert et al., 1998). In contrast, the synthetic progestin, levonorgestrel, appears to antagonize the beneficial impact of oestrogens on endothelial function. In vitro cell work demonstrated that levonorgestrel did not alter endothelial NO synthase mRNA or protein content in human umbilical vein endothelial cells, but estradiol combined with levonorgestrel fully attenuated the increase in endothelial NO synthase mRNA and protein found with estradiol alone, probably decreasing NO bioavailability and thus, capacity for endothelium‐dependent dilatation (Zerr‐Fouineau et al., 2009). Additionally, levonorgestrel is a progestin that is considered androgenic because it has a high binding affinity to androgen receptors in comparison to later‐generation progestins (Sitruk‐Ware, 2005; Toit et al., 2017). Although the effect of testosterone on endothelial function in females is not well understood, these androgenic effects might negatively influence endothelial function in a similar manner to women with polycystic ovary syndrome, who have high testosterone levels and experience impaired FMD (Berbrier et al., 2023).

The group‐level effect of phase in VLD second‐generation OCP users suggests a reproducible impairment of vascular function, now demonstrated across two cycles (present study) and two studies (Torgrimson et al., 2007). In the present study, we tested only young, healthy individuals during days 18–21 of AP exposure, and pooled FMD was 1.1% lower in the AP versus the PP phase across the two cycles. Further research is needed to gain a better understanding of the time course of impairment, whether endothelial impairment is experienced during the entirety of AP exposure, and the magnitude of impairment in all demographics of users. A 1% decrease in FMD is associated with an 8% increased risk of future cardiovascular events (Inaba et al., 2010). Given that OCP users spend 75% of their lifespan in the AP phase, cyclic reductions in endothelial function might have implications for atherosclerotic processes and overall regulation of vascular tone. Longitudinal research is needed to explore the impact of long‐term cyclic reductions in endothelial function with VLD second‐generation OCP use on endothelial function and cardiovascular health.

### 4.2 Individual responses/consistency of responses to the OCP phase

The present study sought to investigate individual responses to OCP phase across two consecutive cycles. Participants were classified as positive, negative or non‐responders to OCP phase using threshold‐based dichotomous classification outlined by Swinton et al. (2018), using a threshold of ±2 × TE. Participants classified with the same response in both cycle 1 and 2 were considered consistent responders. There were three negative responders to phase in each cycle, although these three responses were not consistent (i.e., the participants classified as negative responders in cycle 1 were different participants from the negative responders in cycle 2). The majority of individuals were classified as consistent non‐responders (*n* = 11; 65% of participants), although, as mentioned above, almost half of the ‘consistent’ responses were directionally opposite. The small number of classified negative responders to phase in each cycle despite the group‐level decrease in FMD during the AP phase observed across two OCP cycles indicates that our threshold for dichotomous classification was larger than most changes in FMD observed across phase at the individual level. There are several participants with ΔFMD values approaching but not crossing the threshold for negative response.

The dichotomous classification of responders and non‐responders is contingent on an accurate TE (Swinton et al., 2018). The TE value (used to determine the ±2 × TE threshold) was determined from a separate subset of female participants using a single FMD value from each of the two experimental visits in the low‐hormone phase (EF phase or PP phase) (Tremblay et al., 2019). This differs from the present study because we used the average of three FMD values to provide a single estimate of FMD in each of the four experimental visits. There might be greater day‐to‐day variability when comparing a single measure of FMD rather than an average of three measures. This potentially higher variability would lead to a greater TE and larger threshold. Although there were limited responders to phase, there was no relationship between Δ%FMD in cycles 1 and 2, suggesting that a smaller threshold would not have revealed substantially more consistent responders.

The lack of consistent individual endothelial function responses across two OCP cycles contrasts with our hypothesis. However, these results align with previous findings from our group demonstrating inconsistent intra‐individual FMD responses across the natural menstrual cycle (Liu et al., 2021). Difficulty in predicting the timing of experimental visits across the natural menstrual cycle and inconsistent intra‐individual fluctuations in endogenous estradiol between cycles were thought to contribute to the inconsistent FMD responses across cycles. We hypothesized that consistent doses of synthetic hormones available in a single type and dose of OCPs would allow the detection of consistent individual endothelial function responses over multiple cycles. Contrary to our hypothesis, individual FMD responses to phase with OCP use were also variable, demonstrated by the lack of correlation between the change in FMD in cycles 1 and 2. Although there was a consistent effect of phase on FMD, the lack of consistent individual responses does not support stable, functionally relevant intra‐individual OCP response phenotypes for endothelial function. It is possible that observing individual endothelial function responses across more than two cycles would expose greater overall consistency.

### 4.3 Methodological considerations

The present study included only participants using VLD second‐generation OCPs. This methodological decision was crucial, because the impact of the OCP phase on FMD might differ with OCP type and dose (Williams & MacDonald, 2021). FMD has been reported to decrease during the AP phase in VLD second‐generation OCP users (Torgrimson et al., 2007), to remain stable across phases in LD second‐generation (Torgrimson et al., 2007) and VLD third‐generation OCP users (Thompson et al., 2011), and to increase during the AP phase of LD third‐ and fourth‐generation OCP users (Meendering et al., 2009; Thompson et al., 2011). The inclusion of only one type and dose of OCP ensures that variability in endothelial function responses is not attributable to variable responses of the endothelium to different synthetic hormone compositions. The impact of the OCP phase on FMD across consecutive OCP cycles in third‐ and fourth‐generation OCP users remains an area for further exploration. Although the rigorous control of a population is a strength of this analysis, it also limits the generalizability of the present results. Future research is needed to definitively differentiate the effects of different OCP compositions and their effects on other populations, such as older OCP users or those with greater cardiovascular disease risk.

OCP exposure was controlled with consistent timing of experimental visits during the distinct phases. PP phase experimental visits were scheduled on days 5–7 of the PP week to ensure synthetic hormone washout, because the half‐life of levonorgestrel is ∼20–50 h (Johansson et al., 2002). AP phase experimental visits were on days 5–7 of the third week of APs (end of PP phase) to capture the greatest exposure to the hormone. The timing of OCP ingestion was also controlled to ensure that participants had similar temporal exposure to synthetic hormones. Participants took their OCPs 1–3 h before the experimental visit, because the time to peak concentration of levonorgestrel is 1–4 h (Kook et al., 2002). The temporal effect of synthetic hormones on endothelial function is an area to be explored further. Participants also reported full adherence to their OCPs during the week before coming into the laboratory. There are some recent data suggesting that endogenous and synthetic hormones might not be stable between and within OCP phases (Rodriguez et al., 2024). We did not find a relationship between individual changes in %FMD across phases and changes in endogenous estradiol, progesterone or the oestrogen/progesterone ratio. However, future research should explore the influence of potential individual differences in the metabolism of OCPs and thus systemic synthetic hormone concentrations on FMD across the OCP cycle.

Additionally, we adhered to current methodological guidelines for FMD in order to control for short‐term factors that are known to influence FMD (e.g., physical activity, diet, alcohol, caffeine) (Thijssen et al., 2019). Group‐level responses to the PAR‐Q and PSS support a lack of change in physical activity or stress across experimental visits. Controlling for these factors aimed to limit variability from external factors known to affect FMD. One limitation of the PSS is that baseline stress levels are established based on psychosocial stress experienced over the previous month. This tool might not have the temporal resolution to distinguish differences in stress levels across visits <1 month apart. Further analysis revealed no differences in PSS scores between cycle 1 and 2 AP phase visits (*P* = 0.834) or PP phase visits (*P* = 0.833), which would have fallen 1 month apart, suggesting that stress levels did not change throughout the study.

However, there are several external factors that could have influenced endothelial function that we did not control, such as long‐term diet and sleep patterns. Therefore, these factors could have changed between cycles and influenced the consistency of FMD responses outside the influence of OCP phase. Future studies should aim to assess more factors that could influence the consistency of endothelial responses across two cycles.

## 5 CONCLUSION

This study provides the first observation of the impact of the OCP phase on endothelial function across two consecutive OCP cycles. The results demonstrate that at the group level, VLD second‐generation OCP users experience a decrease in endothelial function with synthetic hormone exposure during the AP phase in comparison to the PP phase. Despite the detection of a group‐level response, analysis of individual responses suggests substantial intra‐individual variability in responses to phase over two OCP cycles. Overall, these results suggest that exposure to the synthetic hormones in second‐generation OCPs might be detrimental to endothelial function, although this was not demonstrated to be a consistent trait‐like response observed at the individual level over two cycles. Given that >150 million individuals use OCPs across the globe (United Nations, 2022), it is essential to continue exploring how long‐term OCP use might impact cardiovascular function and health.

## AUTHOR CONTRIBUTIONS

Lindsay A. Lew and Kyra E. Pyke were responsible for the conception and design of this study. Lindsay A. Lew, Desiree Tugwell, Tess Leavitt and Melanie Vitez collected the data. Lindsay A. Lew and Emily J. Ferguson analysed the data. Lindsay A. Lew and Kyra E. Pyke interpreted the data. Lindsay A. Lew prepared the figures and drafted the manuscript, with revisions from Kyra E. Pyke. All authors approved the final version of the manuscript and agree to be accountable for all aspects of the work in ensuring that questions related to the accuracy or integrity of any part of the work are appropriately investigated and resolved. All persons designated as authors qualify for authorship, and all those who qualify for authorship are listed.

## CONFLICT OF INTEREST

None declared.

## Data Availability

The data that support the findings of this study are available from the corresponding author upon reasonable request.
